# A novel model to predict cancer‐specific survival in patients with early‐stage uterine papillary serous carcinoma (UPSC)

**DOI:** 10.1002/cam4.2648

**Published:** 2019-12-17

**Authors:** Lihua Chen, Xiaona Liu, Mengjiao Li, Shuoer Wang, Hongyu Zhou, Lei Liu, Xi Cheng

**Affiliations:** ^1^ Department of Gynecological Oncology Fudan University Shanghai Cancer Center Shanghai China; ^2^ Department of Oncology Shanghai Medical College Fudan University Shanghai China; ^3^ Shanghai Public Health Clinical Center and Institutes of Biomedical Sciences Fudan University Shanghai China; ^4^ Central Laboratory The Fifth People's Hospital of Shanghai Affiliated to Fudan University Shanghai China

**Keywords:** cancer‐specific death (CSD), competing risk model, nomogram, prediction model, uterine papillary serous carcinoma (UPSC)

## Abstract

**Objective:**

Stage I‐II uterine papillary serous carcinoma (UPSC) has aggressive biological behavior and leads to poor prognosis. However, clinicopathologic risk factors to predict cancer‐specific survival of patients with stage I‐II UPSC were still unclear. This study was undertaken to develop a prediction model of survival in patients with early‐stage UPSC.

**Methods:**

Using Surveillance, Epidemiology, and End Results (SEER) database, 964 patients were identified with International Federation of Gynecology and Obstetrics (FIGO) stage I‐II UPSC who underwent at least hysterectomy between 2004 and 2015. By considering competing risk events for survival outcomes, we used proportional subdistribution hazards regression to compare cancer‐specific death (CSD) for all patients. Based on the results of univariate and multivariate analysis, the variables were selected to construct a predictive model; and the prediction results of the model were visualized using a nomogram to predict the cancer‐specific survival and the response to adjuvant chemotherapy and radiotherapy of stage I‐II UPSC patients.

**Results:**

The median age of the cohort was 67 years. One hundred and sixty five patients (17.1%) died of UPSC (CSD), while 8.6% of the patients died from other causes (non‐CSD). On multivariate analysis, age ≥ 67 (HR = 1.45, *P* = .021), tumor size ≥ 2 cm (HR = 1.81, *P* = .014) and >10 regional nodes removed (HR = 0.52, *P* = .002) were significantly associated with cumulative incidence of CSD. In the age ≥67 cohort, FIGO stage IB‐II was a risk factor for CSD (HR = 1.83, *P* = .036), and >10 lymph nodes removed was a protective factor (HR = 0.50, *P* = .01). Both adjuvant chemotherapy combined with radiotherapy and adjuvant chemotherapy alone decreased CSD of patients with stage I‐II UPSC older than 67 years (HR = 0.47, *P* = .022; HR = 0.52, *P* = .024, respectively). The prediction model had great risk stratification ability as the high‐risk group had higher cumulative incidence of CSD than the low‐risk group (*P* < .001). In the high‐risk group, patients with post‐operative adjuvant chemoradiotherapy had improved CSD compared with patients who did not receive radiotherapy nor chemotherapy (*P* = .037). However, there was no such benefit in the low‐risk group.

**Conclusion:**

Our prediction model of CSD based on proportional subdistribution hazards regression showed a good performance in predicting the cancer‐specific survival of early‐stage UPSC patients and contributed to guide clinical treatment decision, helping oncologists and patients with early‐stage UPSC to decide whether to choose adjuvant therapy or not.

## Graphical abstract

Our prediction model of CSD based on proportional subdistribution hazards regression showed good performance in predicting the cancer‐specific survival of early‐stage UPSC patients and contributes to selection of adjuvant therapy for stage I‐II UPSC patients in clinical treatment strategy.

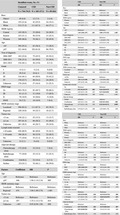

## 1 INTRODUCTION

Endometrial cancer is the most common gynecological malignant tumor and the fourth common cause of cancer death with an increasing incidence rate among women in America.^1^, ^2^ Uterine papillary serous carcinoma (UPSC) belongs to type II endometrial carcinoma. Unlike type I endometrial cancer, UPSC is a non‐hormone dependent tumor. Almost all patients are post‐menopausal elderly women. The onset age of patients with UPSC is 65 to 72 years old, 8 to 10 years older than that of patients with common endometrial cancer,^3^, ^4^ and patients with UPSC were less likely associated with obesity and diabetes. UPSC is a rare histologic subtype of endometrial carcinoma that is highly invasive and extremely liable to occur extrauterine spreading and lymph node metastasis. UPSC makes up about 10% of endometrial cancer cases, but leads to 39% of all endometrial cancer deaths.^5^ The 5‐year survival for UPSC ranges from 50% to 80% compared with 80 to 90% for endometroid cancer.^6^ Even early‐stage UPSC has a high recrudesce rate and a poor prognosis. As a biologically aggressive subtype of Type II endometrial cancer, whether the clinicopathological risk factors like age, grade, disease stage and lymph vascular space invasion (LVSI) in Type I carcinomas suit for UPSC is undefined.^7^ Some small‐cohort studies have explored the prognostic factors for clinical outcomes in UPSC,^8^, ^9^ and a straightforward prediction model based on large‐scale population for UPSC is still unknown. Therefore, our study aimed to develop an explicit prognostic model based on proportional subdistribution hazards regression which predicts the cancer‐specific death for UPSC patients by analyzing the Surveillance, Epidemiology, and End Results (SEER) database.

## 2 METHODS

### 2.1 Data extraction

**Figure 1:**
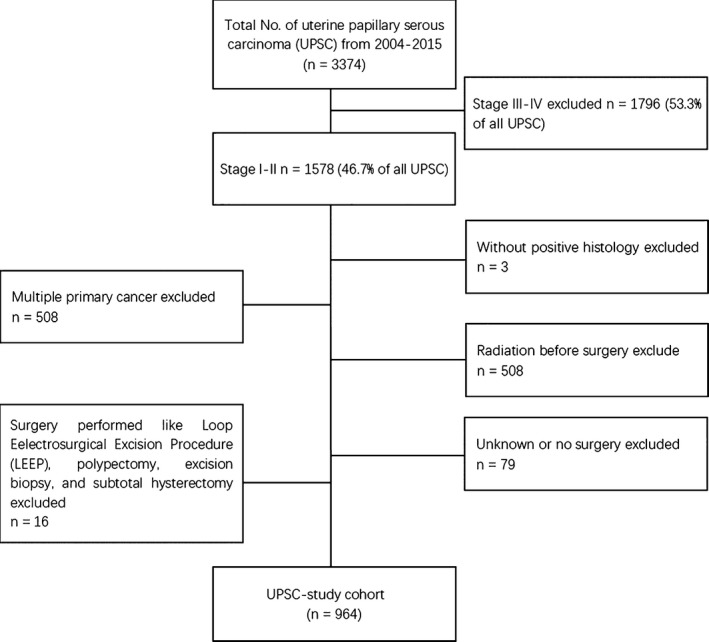
Flow chart of patient selection from the Surveillance, Epidemiology, and End Results (SEER) database

SEER is a population‐based cancer registry maintained by the National Cancer Institute. Three thousand three hundred and seventy four patients whose histology was diagnosed as UPSC from January 2004 to December 2015 were found in the database by using SEER*Stat 8.3.2 software. Patients with stage III‐IV UPSC and patients had no positive histology were excluded. UPSC patients who were combined with other primary malignant tumors were also foreclosed. Patients who had received preoperative radiotherapy and had no surgery or had inadequate surgery such as Loop Eelectrosurgical Excision Procedure (LEEP), polypectomy, subtotal hysterectomy with cervix preserved were eliminated. All patients enrolled in the cohort underwent at least total hysterectomy. A total of 964 patients with stage I‐II UPSC were selected for further analysis (Figure 1). SEER summary staging (localized, regional, distant, and unknown) was used to categorize the extent of the disease as a surrogate for the traditional FIGO staging and defined by the derived SEER Summary Stage 2000 variable. The SEER staging system corresponds to the commonly used International Federation of Gynecology and Obstetrics (FIGO) staging system in the following way: localized (FIGO IA, IB, I‐not otherwise specified [NOS]), regional (II, III‐A, III‐B, III‐NOS), and distant (FIGO IV‐A, IV‐B, IV‐NOS).^10^

### 2.2 Statitical methods

#### 2.2.1 Proportional subdistribution hazards regression

Although the Cox proportional hazards regression model is the most common method for analyzing survival data, it tends to overrate the risk of the disease when there are competing risk events, which leads to inaccurate estimates consequently. This is because the competing risks of the event of interest were treated as censored in the Cox proportional hazards regression model. As most UPSC patients are post‐menopausal elderly women, whose median age in our study was 67 years, competing risks are especially relevant in the study of UPSC. To obtain unbiased estimates of the risk of cancer‐specific death for UPSC, we used a proportional subdistribution hazards regression, which connects the regression coefficients to a cumulative incidence function.^11^

#### 2.2.2 Variable selection and model development

Combined with the results in the univariate and multivariate analysis, the following variables were considered for predicting the risk of cancer‐specific death for UPSC: age at UPSC diagnosis, grade, FIGO, SEER summary stage tumor size, and regional lymph nodes examined. To select variables to be included in the final prediction model, we used stepwise forward and backward elimination methods. In each step, a variable was considered for addition to or subtraction from the set of variables on the basis of some prespecified criterion. We used the Bayesian information criteria (BIC) as criteria for the selection of a model.^12^ Three variables were selected into the final model including age, SEER summary stage and tumor size.

#### 2.2.3 Performance evaluation of prediction model

In order to evaluate the performance of the prediction model, we used the c‐index (index of concordance). To further examine the capability of the prediction model, the risk score was calculated for each patient and the median risk score was used as the cutoff to classify patients into high‐risk groups and low‐risk groups. We then estimated observed cumulative incidence using the Gray method for each group by considering competing risks to see the risk stratification ability of the prediction model. The risk score was calculated as follows:

$$\text{Risk score}=\sum_{i=1}^{n}\beta i*xi$$

where *βi* indicates the regression coefficient and *xi* refers to the assigned value of the corresponding factor in the model.

#### 2.2.4 Nomogram

In order to visualize the results, the nomogram was developed based on the competing risk regression model to describe the individual probability of CSD. Assigned a value to each factor according to its contribution degree to the outcome variable in the model (the regression coefficient), then added each part to get the total score. Through a function conversion, thereby calculating a predicted probability of the individual outcome event for each subject.

All statistical analyses were conducted using R (https://www.r-project.org/).

## 3 RESULTS

### 3.1 Patient demographics

A total of 964 patients with FIGO stage I‐II were identified. The median follow‐up period of stage I‐II UPSC in this study was 46 months (range, 0‐143 months). The median age of the cohort was 67 years. 17.1% (n = 165) of the patients died of UPSC (CSD), while 8.6% of the patients died from other causes (non‐CSD). Most patients were diagnosed with stage IA (n = 635, 65.9%). Due to the limited number of patients with stage IB and stage II UPSC, stage IB and stage II UPSC patients are merged in the subsequent analysis. Overall, 254 patients (26.3%) were treated with a combination of radiotherapy and chemotherapy, 211 patients (21.9%) were treated with chemotherapy alone, 123 patients (12.8%) were treated with radiotherapy alone and 376 patients (39.0%) were treated with neither chemotherapy nor radiotherapy. Eight hundred and three patients (83.3%) had at least one lymph node resected (Table 1).

**Table 1:** Patient demographics of study population and association between patient chaeacteristics and CSD

	Stratified events, No. (%)		
	Censored	CSD	Non‐CSD
	N = 716 (74.3)	N = 165 (17.1)	N = 83 (8.6)
Race			
Others^a^	49 (6.8)	12 (7.3)	3 (3.6)
Black	155 (21.6)	42 (25.5)	16 (19.3)
White	512 (71.5)	111 (67.3)	64 (77.1)
SEER registry			
Central	145 (20.3)	33 (20.0)	24 (28.9)
Eastern	223 (31.1)	56 (33.9)	21 (25.3)
Western	348 (48.6)	76 (46.1)	38 (45.8)
Age			
<67	395 (55.2)	63 (38.2)	17 (20.5)
≥67	321 (44.8)	102 (61.8)	66 (79.5)
Year of diagnosis			
2004‐2007	162 (22.6)	75 (45.5)	45 (54.2)
2008‐2011	238 (33.2)	64 (38.8)	32 (38.6)
2012‐2015	316 (44.1)	26 (15.8)	6 (7.2)
Grade			
I	15 (2.1)	4 (2.4)	0 (0.0)
II	39 (5.4)	10 (6.1)	3 (3.6)
III	316 (44.1)	84 (50.9)	35 (42.2)
IV	170 (23.7)	40 (24.2)	24 (28.9)
Unknown	176 (24.6)	27 (16.4)	21 (25.3)
FIGO stage			
IA	503 (70.3)	82 (49.7)	50 (60.2)
IB	81 (11.3)	31 (18.8)	17 (20.5)
II	78 (10.9)	34 (20.6)	9 (10.8)
INOS	54 (7.5)	18 (10.9)	7 (8.4)
SEER summary stage			
Localized	596 (83.2)	111 (67.3)	65 (78.3)
Regional	120 (16.8)	54 (32.7)	18 (21.7)
Tumor size			
<2 cm	158 (22.1)	25 (15.2)	13 (15.7)
≥2 cm	297 (41.5)	96 (58.2)	41 (49.4)
Unknown	261 (36.5)	44 (26.7)	29 (34.9)
Lymph nodes resected			
>10 nodes	436 (60.9)	66 (40.0)	35 (42.2)
1‐10 nodes	188 (26.3)	56 (33.9)	22 (26.5)
No	77 (10.8)	35 (21.2)	26 (31.3)
Unknown	15 (2.1)	8 (4.8)	0 (0.0)
Adjuvant therapy			
Chemotherapy alone	172 (24.0)	32 (19.4)	7 (8.4)
Radiotherapy alone	77 (10.8)	25 (15.2)	21 (25.3)
Combination	216(30.2)	32 (19.4)	6 (7.2)
Neither	251(35.1)	76 (46.1)	49 (59.0)

Abbreviations: CSD, cancer specific death; Non‐CSD, non‐cancer specific death.

a Others: including American Indian/Alaska Native, Asian or Pacific Islander

### 3.2 Survival analysis based on competing risk regression model

**Figure 2:**
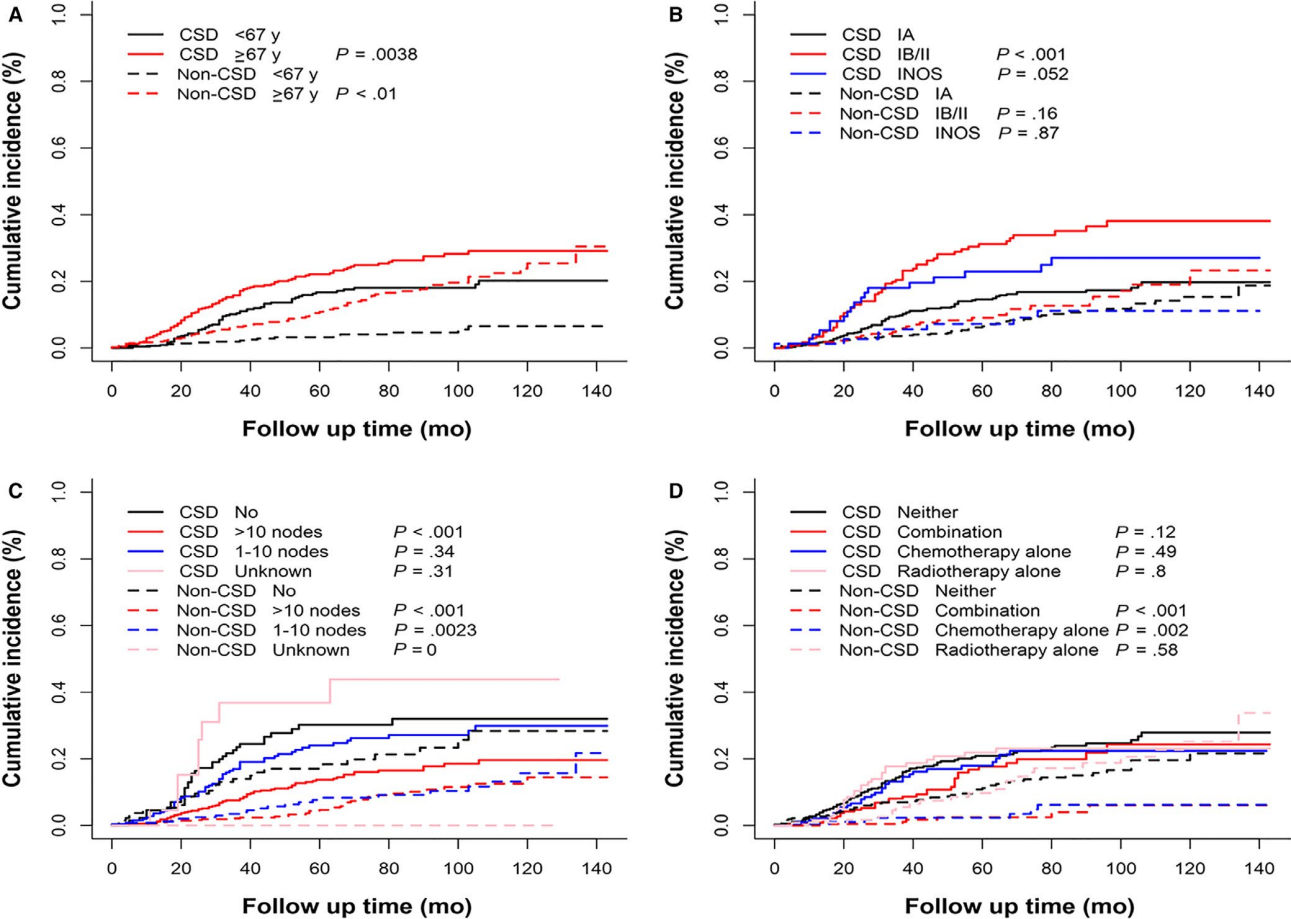
The cumulative incidence curve of CSD and non‐CSD of patients with stage I‐II UPSC using Gray method. A, Patients who were older than 67 years had higher cumulative incidence of CSD and non‐CSD than their counterparts. (*P* = .0038, *P* < .01, respectively). B, Patients with stage IB/II UPSC had higher cumulative incidence of CSD than stage IA patients (*P* < .001). C, Patients who had >10 lymph nodes resected showed a lower cumulative incidence of CSD (*P* < .001) than patients who had no node removed. D, Patients who were treated with a combination of chemotherapy and radiotherapy (*P* < .001) or chemotherapy alone (*P* = .002) had lower cumulative incidence of non‐CSD than patients who were not treated with chemotherapy or radiotherapy

In the univariate analysis based on Gray method, patients whose age were ≥67 had higher cumulative incidence of CSD and non‐CSD than that of its counterpart. (*P* = .0038, *P* < .001, respectively) (Figure 2A). Stage IB/II UPSC patients had higher cumulative incidence of CSD than stage IA UPSC patients (*P* < .001)(Figure 2B). Patients who had >10 lymph nodes removed showed lower cumulative incidence of CSD (*P* < .001) than patients with no nodes (Figure 2C). Patients who were treated with a combination of chemotherapy and radiotherapy or chemotherapy alone had lower cumulative incidence of non‐CSD (*P* < .001, *P* = .0022, respectively) than patients who were treated with neither chemotherapy nor radiotherapy. However, CSD of these patients did not improve (Figure 2D). In the multivariate analysis based on proportional subdistribution hazards regression, age ≥ 67 years (HR = 1.45, *P* = .021) and tumor size ≥2 cm (HR = 1.81, *P* = .014) were risk factors of CSD for stage I‐II UPSC patients while >10 lymph nodes (HR = 0.52, *P* = .003) resected was a protective factor of CSD for stage I‐II UPSC patients. Patients who were older than 67 years (HR = 3.08, *P* < .001) had higher risk of non‐CSD, and patients who underwent lymphadenectomy (>10 nodes resected, HR = 0.44, *P* = .003; 1‐10 nodes, HR = 0.47, *P* = .012) had less non‐CSD than patients who had no nodes removed. Use of a combination of chemotherapy and radiotherapy (HR = 0.26, *P* = .002) and use of chemotherapy alone (HR = 0.32, *P* = .005) were favorable factors for non‐CSD of the patients with stage I‐II UPSC but not for CSD. There were no statistically significant differences in race, SEER registry, year of diagnosis, histology grade, FIGO stage, SEER summary stage and radiotherapy subgroups toward both CSD and non‐CSD (Table 2). In the subgroup analysis stratified by age, patients with stage IB/II (HR = 1.83, *P* = .036) UPSC had higher risk of CSD than those with stage I and >10 lymph nodes resected (HR = 0.50, *P* = .010) still was a protective factor for CSD in patients who were older than 67 years. Use of a combination of chemotherapy and radiotherapy (HR = 0.47, *P* = .022) and use of chemotherapy alone (HR = 0.52, *P* = .024) were associated with decreased CSD in age ≥67 years group. Lymphadenectomy (>10 lymph nodes resected, HR = 0.42, *P* = .004; 1‐10 nodes, HR = 0.48, *P* = .031) and use of a combination of chemotherapy and radiotherapy (HR = 0.19, *P* = .007) were associated with decreased non‐CSD (Table 3).

**Table 2:** Multivariate analysis

	CSD		Non‐CSD	
	HR	*P*	HR	*P*
Race				
White	Reference		Reference	
Black	1.46 (0.99‐2.16)	.054	1.00 (0.56‐1.80)	1.000
Other	1.02 (0.53‐1.97)	.950	0.84 (0.26‐2.68)	.820
SEER registry				
Western	Reference		Reference	
Central	0.84 (0.55‐1.27）	.410	1.42 (0.83‐2.42)	.200
Eastern	1.17 (0.81‐1.71）	.410	1.11 (0.62‐1.98)	.720
Age				
<67	Reference		Reference	
≥67	**1.45 (1.06‐1.99）**	**.021**	**3.08 (1.75‐5.43)**	**<.001**
Year of diagnosis				
2004‐2007	Reference		Reference	
2008‐2011	0.83 (0.59‐1.17）	.300	1.10 (0.67‐1.79)	.700
2012‐2015	0.83 (0.51‐1.34）	.440	0.55 (0.23‐1.30)	.170
Grade				
I/II	Reference		Reference	
III	1.03 (0.57‐1.89)	.900	2.55 (0.80‐8.09)	.110
IV	0.93 (0.49‐1.78)	.830	3.07 (0.95‐9.91)	.060
Unknown	0.72 (0.37‐1.41)	.340	2.63 (0.77‐8.98)	.120
FIGO stage				
IA	Reference		Reference	
IB/II	1.53 (0.98‐2.40)	.059	1.24 (0.66‐2.31)	.500
INOS	0.98 (0.52‐1.86)	.960	0.93 (0.34‐2.54)	.890
SEER summary stage				
Localized	Reference		Reference	
Regional	1.44 (0.91‐2.30)	.120	0.82 (0.40‐1.68)	.590
Tumor size				
<2CM	Reference		Reference	
≥2CM	**1.81 (1.13‐2.90)**	**.014**	1.55 (0.83‐2.88)	.170
Unknown	0.97 (0.58‐1.60)	.900	1.20 (0.64‐2.26)	.570
Lymph nodes resected				
No	Reference		Reference	
>10 nodes	**0.52 (0.34‐0.79)**	**.002**	**0.44 (0.26‐0.75)**	**.003**
1‐10 nodes	0.83 (0.53‐1.29)	.410	**0.47 (0.26‐0.85)**	**.012**
Unknown	1.47 (0.64‐3.44)	.380	0 (0‐0)	<.001
Adjuvant therapy				
Neither	reference		reference	
Combination	0.65 (0.41‐1.02)	.630	**0.26 (0.11‐0.61)**	**.002**
Chemotherapy alone	0.77 (0.51‐1.18)	.230	**0.32 (0.14‐0.71)**	**0.005**
Radiotherapy alone	0.76 (0.47‐1.23)	.260	1.03 (0.60‐1.78)	.920

Abbreviations: CSD, cancer specific death; Non‐CSD, non‐cancer specific death. Bold values indicates statistically significant difference.

**Table 3:** Multivariate analysis for age ≥ 67 years cohort

	CSD		Non‐CSD	
	HR	*P*	HR	*P*
Race				
White	Reference		Reference	
Black	1.36 (0.829‐2.27)	.230	1.14 (0.58‐2.27)	.710
Other	0.44 (0.09‐2.02)	.290	1.27 (0.37‐4.40)	.710
SEER registry				
Western	Reference		Reference	
Central	1.17 (0.70‐1.96)	.560	1.16 (0.62‐2.16)	.640
Eastern	1.44 (0.88‐2.34)	.140	1.11 (0.57‐2.15)	.760
Year of diagnosis				
2004‐2007	Reference		Reference	
2008‐2011	0.82 (0.53‐1.30)	.400	0.87 (0.49‐1.54)	.630
2012‐2015	0.66 (0.33‐1.30)	.230	0.70 (0.29‐1.72)	.440
Grade				
I/II	Reference		Reference	
III	1.47 (0.60‐3.59)	.400	2.14 (0.68‐6.73)	.190
IV	0.93 (0.35‐2.48)	.890	2.00 (0.62‐6.49	.250
Unknown	1.14 (0.45‐2.86)	.780	1.52 (0.44‐5.27)	.510
FIGO stage				
IA	Reference		Reference	
IB/II	**1.83 (1.04‐3.21)**	**.036**	0.91 (0.46‐1.80)	.780
INOS	1.32 (0.59‐2.96)	.500	0.76 (0.21‐2.70)	.670
SEER summary stage				
Localized	Reference		Reference	
Regional	1.44 (0.81‐2.56)	.210	0.92 (0.39‐2.21)	.860
Tumor size				
<2 cm	Reference		Reference	
≥2 cm	1.80 (0.98‐3.31)	.057	1.37 (0.68‐2.75)	.380
Unknown	0.75 (0.38‐1.46)	.390	1.23 (0.60‐2.50)	.570
Lymph nodes resected				
No	Reference		Reference	
>10 nodes	**0.50 (0.29‐0.85)**	**.010**	**0.42 (0.23‐0.76)**	**.004**
1‐10 nodes	0.71 (0.41‐1.24)	.230	**0.48 (0.24‐0.93)**	**.031**
Unknown	1.31 (0.46‐3.73)	.610	0(0‐0)	<.001
Adjuvant therapy				
Neither	Reference		Reference	
Combination	**0.47 (0.25‐0.90)**	**.022**	**0.19 (0.06‐0.64)**	**.007**
Chemotherapy alone	**0.52 (0.30‐0.92)**	**.024**	0.44 (0.19‐1.04)	.060
Radiotherapy alone	0.69 (0.39‐1.23)	.210	1.16 (0.62‐2.16)	.650

Abbreviations: CSD, cancer specific death; Non‐CSD, non‐cancer specific death. Bold values indicates statistically significant difference.

### 3.3 Prognostic model development and evaluation

**Figure 3:**
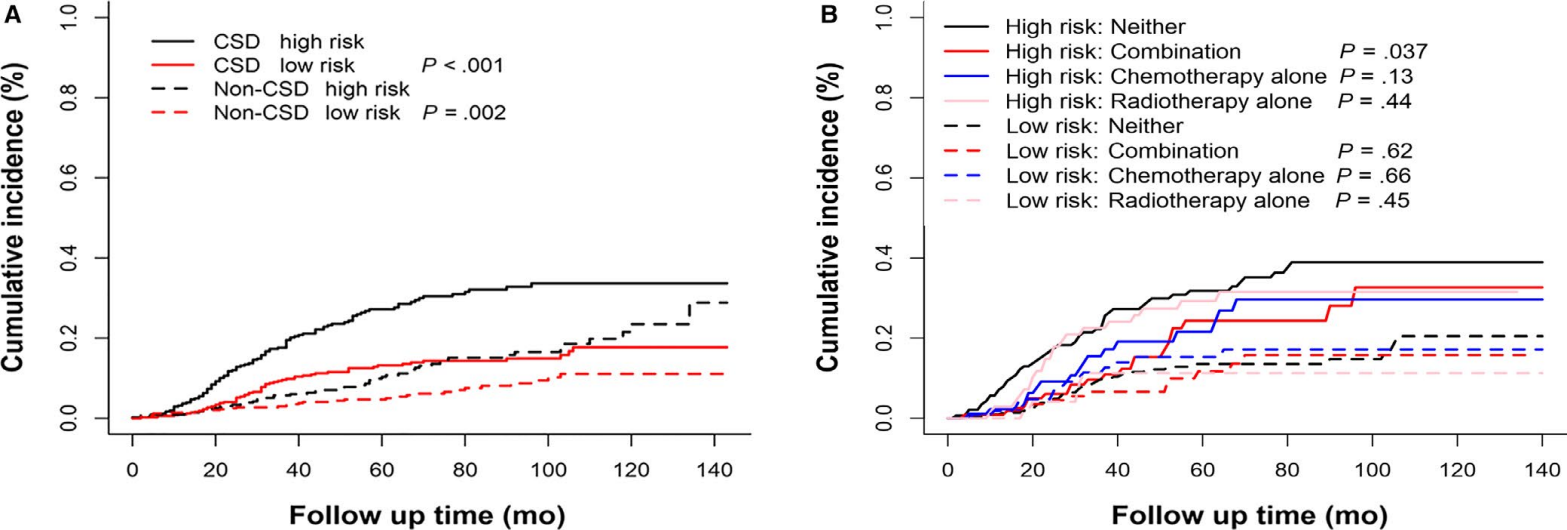
The stratification ability of the prediction model and the relationship between the model and adjuvant therapy. A, By calculating the risk score for each patient based on the model, patients were classified into the high‐risk group and low‐risk group. The high‐risk group was related to increased CSD compared with the low‐risk group (*P* < .001). B, Patients treated with a combination of chemotherapy and radiotherapy showed improved cancer‐specific survival in the high‐risk group (*P* = .037) while there was no such effect in the low‐risk group

**Figure 4:**
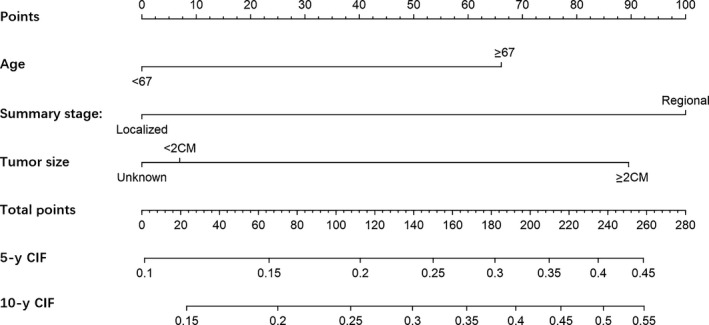
The nomogram of the model for predicting the probability of cancer‐specific death (CSD) in early‐stage UPSC patients

In order to select the variables to build prediction model, we used stepwise forward and backward elimination methods based on the significantly different variables in the mutivariate regression analysis to identify the variables which performed well in the prognostic model. The final selected variables were listed in Table 4 and the results indicated that the age of patients when UPSC diagnosed, SEER summary stage of UPSC and tumor size were significantly associated with CSD. The regression coefficients in the competing risk model and the value of the variables were used to calculate the risk score for each patient, then the median risk score was used as the cutoff to classify patients into high‐risk group and low‐risk group. As is shown in Figure 3A, the group of high risk was associated with higher cumulative incidence of CSD (*P* < .001) than the low‐risk group which indicates that the prognostic model we constructed in this study performed well. The c‐index of the model is 0.643, with moderate discriminatory power. In order to validate the performance of the prediction model, patients were stratified by risk score. Interestingly, use of a combination of chemotherapy and radiotherapy was significantly associated with improved CSD (HR = 0.58, *P* = .037) in the high‐risk group but not in the low‐risk group (Figure 3B). Furthermore, we built a nomogram based on the prediction model to predict the probability of CSD for every individual. The plot of the early‐stage UPSC nomogram is shown in Figure 4. The calibration plots presented excellent agreement between the nomogram prediction and the actual observation for the 5‐, and 10‐year cumulative incidences (Figure S1).

**Table 4:** The selected variables for model construction

Factors	Coefficient	HR	*P*
Age			
<67	Reference	Reference	Reference
≥67	.446	1.56 (1.14‐2.14)	.005
SEER summary stage			
Localized	Reference	Reference	Reference
Regional	.674	1.96 (1.40‐2.74)	<.001
Tumor size			
<2 cm	Reference	Reference	Reference
≥2 cm	.557	1.75 (1.12‐2.72)	.014
Unknown	−.047	0.95 (0.58‐1.56)	.850

## 4 DISCUSSION

The analysis suggested that age ≥ 67 years and tumor size ≥ 2 cm were associated with increased CSD of early‐stage UPSC patients, while >10 lymph nodes removed was correlated with improved CSD. It was not surprising that age ≥ 67 years was also associated with higher cumulative incidence of non‐CSD as elderly UPSC patients had other comorbities such as cardiovascular and cerebrovascular diseases. However, use of a combination of chemotherapy and radiotherapy was related to decreased non‐CSD rather than CSD. The most likely explanation is that patients who received chemotherapy were in better physical status than the patients who received no chemotherapy were. Therefore, patients who received chemotherapy were more likely to have lower incidence of non‐CSD.^13^ When the patients were stratified by age, FIGO stage IB/II UPSC patients had worse CSD compared with FIGO stage IA UPSC patients in the age ≥ 67 years group. In this group, >10 lymph nodes resected was the favorable factor for CSD and the recipients of a combination of chemotherapy and radiotherapy as well as the recipients of chemotherapy alone had better cancer‐specific survival than the patients who were not treated with chemotherapy nor radiotherapy. In our study, we constructed a prediction model to forecast cancer‐specific survival for early‐stage UPSC patients based on proportional subdistribution hazards regression. By applying risk score, we divided patients into high‐risk and low‐risk group, and we observed that patients in the high‐risk group had increased CSD compared with the low‐risk group, which indicated that the prognostic model performed well. Furthermore, use of a combination of chemotherapy and radiotherapy was significantly correlated with improved cancer‐specific survival compared with patients who underwent no adjuvant therapy in the high‐risk group, while there was no such effect in the low‐risk group. It was suggested that calculating risk score based on our prediction model could help oncologists to determine whether the early stage UPSC patients received a combination of adjuvant chemotherapy and radiotherapy or not. Considering chemoradiotherapy may also have a significant impact on quality of life of patients, it is important that low‐risk patients could aviod unnessary chemotherapy via risk stratification.^14^

Since the survival data of cancer patients are often accompanied by multiple outcomes and a competitive relationship existed between the outcomes, the competing risk model is increasingly used in survival analysis. While traditional Cox proportional hazards regression treats competing events as censored data which may falsely evaluate the effects on survival of covariate, the competing risk model provided a novel method to analyze cancer‐specific death. Dan Li et al reported competing nomograms based on competing risk model helped in the selection of adjuvant therapy for elderly colon cancer patients, considering elderly colon cancer patients often have competing issues that might affect their life expectancy and cancer outcomes.^13^ Summer S. Han et al built a prediction model to evaluate the risk of second primary lung cancer (SPLC) among survivors with initial primary lung cancer (IPLC).^15^ Due to the rarity of UPSC, especially early‐stage UPSC, limited prospective clinical trials were reported to study on the adjuvant therapy to guide the postoperative treatment for stage I‐II UPSC patients. In our study, we noted that use of a combination of chemotherapy and radiotherapy was not associated with cancer‐specific survival benefit in the whole stage I‐II UPSC cohort via multivariate analysis. But when the patients were stratified by age, the recipients of a combination of chemotherapy and radiotherapy had improved survival outcome in age ≥ 67 years group. Some small studies have suggested that women with early‐stage UPSC benefited from adjuvant therapy with improved progression‐free survival (PFS) and overall survival (OS), particularly in stage IB and stage II patients.^6^, ^8^, ^16^ Amanda Nickles Fader et al reported that adjuvant chemotherapy should be considered for early‐stage UPSC patients irrespective of age in a retrospective study (n = 206).^8^ Recently, a population‐based study utilizing data from the National Cancer Data Base (NCDB) concluded that early‐stage UPSC patients benefited from postoperative chemotherapy, particularly those with stage IB and II neoplasms. However, the database captured the overall mortality rate of patients but the cancer‐specific survival of patients was unavailable.^17^ Other studies draw a conclusion that chemotherapy failed to improve survival in early stage UPSC patients due to the limited number of patients who received chemotherapy or the interference of other unbalanced cofounders.^18^, ^19^, ^20^, ^21^ A small study (n = 55) showed that use of chemotherapy had no significant effect on OS in patients with stage II UPSC, possibly because the sample size was too small and lacked statistical power to study OS.^22^ Therefore, we built a prediction model based on proportional subdistribution hazards regression and calculated the risk score for each patient by using regression coefficient and the assigned value of the variable in the model. By using this prediction model, stage I‐II UPSC patients can be divided into either high‐risk cohort or low‐risk cohort. Use of a combination of chemotherapy and radiotherapy was associated with remarkably improved survival in high‐risk group while there was no such effect in the low‐risk group. As patients in the high‐risk group are more likely to have worse CSD, post‐operative adjuvant chemoradiotherapy should be administered and patients should be more closely followed. In contrast, patients in the low‐risk group can avoid unnecessary treatment.

The role of radiotherapy alone for UPSC patients is still unknown. Some studies reported that radiotherapy enhanced local control rate of pelvic but did not contributed to overall survival, since UPSC patients could have extrapelvic metastasis even early in the disease.^16^, ^23^, ^24^ In this study, we noted a similar finding that radiotherapy alone was not associated with cancer‐specific survival regardless of the form of radiation. This may be due to the fact that the radiotherapy information has been underascertained in the SEER database.^25^

Consistent with the previous study,^21^ black women with UPSC tended to have worse survival compared to white women in the multivariate analysis (HR = 1.46, *P* = .054), but it was not significant possibly due to the limited number of black women in this study (n = 213).

Although our study has certain strengths, we acknowledge several limitations. First, although the SEER database captures data on the use of chemotherapy, the explicit agents utilized, number of cycles, and timing was not recorded. Similarly, treatment fields and information on dosing were lacking for those who received radiation. Second, we could not explore the relationship between risk factors and recurrence free survival (RFS) because of no recurrence information of patients in the SEER database. Finally, the levels of CA125 were not recorded for UPSC in the SEER database as some studies reported that CA125 could be regarded as a prediction factor for survival. Analogously, many of the results of peritoneal cytology were recorded as unknown thus affecting survival analysis.

In summary, our study investigated the clinicopathological factors associated with cancer‐specific death in early‐stage UPSC patients and suggested that use of a combination of chemotherapy and radiation or use of chemotherapy alone was correlated with improved CSD in the age ≥ 67 years group by analyzing the SEER database. We also developed a prognostic model based on the competing risk model and by utilizing the risk score derived from the model, we classified the patients into high‐risk and low‐risk groups. We found that use of chemoradiotherapy was associated with better survival particularly in high‐risk group. We also calculated the cumulative incidence of cancer‐specific death for each patients using the developed nomogram. Nomogram made the prediction model more friendly to oncologists and helped oncologists select early‐stage UPSC patients who might benefit from chemoradiotherapy.

## 5 CONCLUSION

Our prediction model of CSD based on proportional subdistribution hazards regression showed a good performance in predicting the cancer‐specific survival of early‐stage UPSC patients and contributed to guide clinical decision‐making about whether stage I‐II UPSC patients could choose a combination of adjuvant chemotherapy and radiotherapy or not.

## CONFLICT OF INTEREST

The authors report no conflict of interest this work.

## Data Availability

The data that support the findings of this study are available in the Surveillance, Epidemiology, and End Results (SEER) database at https://seer.cancer.gov/.
