# Supplementary figures and images for: A novel model to predict cancer‐specific survival in patients with early‐stage uterine papillary serous carcinoma (UPSC)
Source: Cancer Med. 2019 Dec 17;9(3):988–98. doi: 10.1002/cam4.2648 (PMC6997089; doi:10.1002/cam4.2648)

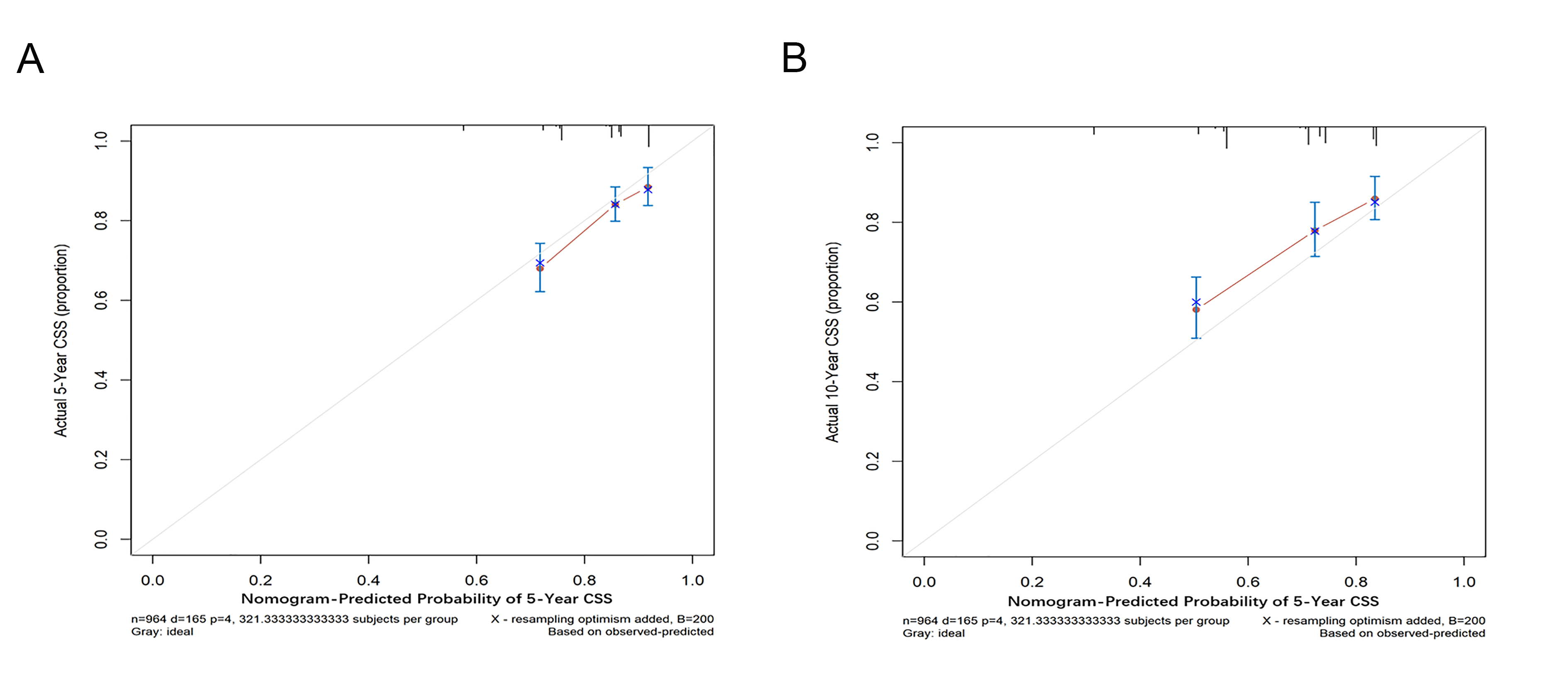

Supplement: Supplementary file 1 [file CAM4-9-988-s001.tif]
